# Interactions of Terahertz Photons with Phonons of Two-Dimensional van der Waals MoS_2_/WSe_2_/MoS_2_ Heterostructures and Thermal Responses

**DOI:** 10.3390/ma18071665

**Published:** 2025-04-04

**Authors:** Jingwen Huang, Ningsheng Xu, Yumao Wu, Xue Ran, Yue Fang, Hongjia Zhu, Weiliang Wang, Huanjun Chen, Shaozhi Deng

**Affiliations:** 1 State Key Laboratory of Optoelectronic Materials and Technologies, Guangdong Province Key Laboratory of Display Material and Technology, School of Electronics and Information Technology, Sun Yat-sen University, Guangzhou 510275, Chinazhuhj3@mail2.sysu.edu.cn (H.Z.); 2 State Key Laboratory of Integrated Chips and Systems, Frontier Institute of Chip and System, Fudan University, Shanghai 200433, China; 3 State Key Laboratory of Integrated Chips and Systems, School of Information Science and Technology, Fudan University, Shanghai 200433, China; yumaowu@fudan.edu.cn (Y.W.); xran24@m.fudan.edu.cn (X.R.); 4 Guangdong Province Key Laboratory of Display Material and Technology, Center for Neutron Science and Technology, School of Physics, Sun Yat-sen University, Guangzhou 510275, Chinawangwl2@mail.sysu.edu.cn (W.W.)

**Keywords:** van der Waals MoS_2_/WSe_2_/MoS_2_ heterostructures, terahertz time-domain spectroscopy, low-wavenumber Raman spectroscopy, terahertz phonon spectroscopy, photon-phonon interactions, photothermal effects

## Abstract

The interaction between terahertz (THz) photons and phonons of materials is crucial for the development of THz photonics. In this work, typical two-dimensional (2D) van der Waals (vdW) transition metal chalcogenide (TMD) layers and heterostructures are used in THz time-domain spectroscopy (TDS) measurements, low-wavenumber Raman spectroscopy measurements, calculation of 2D materials’ phonon spectra, and theoretical analysis of thermal responses. The TDS results reveal strong absorption of THz photons in the frequency range of 2.5–10 THz. The low-wavenumber Raman spectra show the phonon vibration characteristics and are used to establish phonon energy bands. We also set up a computational simulation model for thermal responses. The temperature increases and distributions in the individual layers and their heterostructures are calculated, showing that THz photon absorption results in significant increases in temperature and differences in the heterostructures. These give rise to interesting photothermal effects, including the Seebeck effect, resulting in voltages across the heterostructures. These findings provide valuable guidance for the potential optoelectronic application of the 2D vdW heterostructures.

## 1. Introduction

Terahertz waves are electromagnetic radiation with a frequency range of 0.1–10 THz and a wavelength of 0.03–3 mm. Due to their frequency band lying between the radio wave region and the optical region, they possess dual characteristics of both macroscopic classical theory and microscopic quantum theory, thus exhibiting unique application value [1,2]. The unique frequency range of THz spectroscopy (covering characteristic frequencies of molecular vibrations and lattice vibrations) makes it a powerful tool for characterizing the physical properties of materials, such as measuring complex permittivity, dynamic conductivity, and studying microscopic processes like low-energy electron transitions and carrier dynamics. Furthermore, THz technology demonstrates broad application prospects in multiple cutting-edge fields, including biomedical imaging, security inspection, 6G communications, and materials science [3,4,5,6,7]. Its potential for high-frequency communications is especially regarded as one of the core technologies to break through the communication rate bottleneck in the future [8,9,10].

The main challenges and development directions of THz technology lie in achieving high spatial resolution, broadband spectral coverage, high signal-to-noise ratio, high efficiency, and system integrability. In this context, exploring emerging materials, such as 2D materials, heterostructures, and integration methods, is attracting increasing attention [11,12].

Two-dimensional materials have the advantage of being easily integrated with traditional optoelectronic materials, systems, and devices, bringing novel phenomena and potential applications in the THz field [13,14]. In particular, the vdW heterostructures are stacked from different layered materials, with layers bound together by vdW forces, free from the constraints of lattice matching between the material and the substrate. At the same time, since electrons in atomically thin layers are directly exposed, different quantum states between layers can interact and couple in ways that are not possible in other systems, enabling the ultrafast transfer of electrons and holes across layers within an atomically spaced distribution. Compared to individual 2D atomic crystal materials, 2D vdW heterostructures exhibit significantly enhanced performance advantages over monolayer materials [15,16,17].

The research directions regarding the coupling between THz photons and phonons in 2D materials, such as black phosphorus, MoS_2_, and WSe_2_, involve several aspects [18,19,20]. (1) Phonon polaritons: the coupling of THz photons with phonons to form quasiparticles. (2) Thermal effects and energy conversion: THz wave energy dissipation due to the conversion of electromagnetic wave energy into lattice vibrations, i.e., the phonons. (3) Nonlinear optical response: nonlinear effects of phonons induced by strong-field THz pulses. Phonons, as the quantized modes of atomic oscillations within a lattice, play a crucial role in the thermal and electrical properties of materials. Optical phonons can be generated through optical excitation, and manipulating phonons in semiconductors allows for the control of material properties at the quantum level. Traditional methods for phonon excitation typically rely on the illumination of the materials using infrared or visible light sources. However, despite the unique characteristics of THz radiation, the direct excitation of coherent optical phonons has faced the challenge of energy mismatch.

Currently, there is limited research on the interactions between photons and phonons of transition metal dichalcogenide (TMD) materials and their heterostructures. It is mainly because monolayer TMD materials, such as MoS_2_, exhibit extremely high transmittance to THz waves, and as the incident wave frequency increases, the absorption rate of THz waves gradually decreases [21,22,23,24]. Zhu et al. [25] fabricated a broadband THz wave absorber based on MoS_2_. Through phase modulation, broadband coherent absorption can be achieved, with the coherent absorption rate being adjustable from 1.57% to 99.97%. This research result indicates that with certain scientific modulation methods, TMD materials can also exhibit excellent absorption performance for THz wave radiation. H. Buss et al. [26] studied the broadband transient conductivity and the low-energy excitations in multilayer MoS_2_. When the sample was subjected to radiation with a frequency exceeding 2 THz at low temperatures, a significant enhancement in conductivity was observed. This temperature-dependent behavior suggests that multilayer MoS_2_ possesses strong high-frequency optoelectronic responses in the THz regime, particularly at frequencies above 2 THz.

In this work, 2D vdW MoS_2_/WSe_2_/MoS_2_ heterostructures with high-quality atomic-level crystalline interfaces were fabricated using an optical micromechanical arm alignment technique. THz time-domain spectroscopy measurements revealed significant absorption of different structurally layered materials in the range of 0.5 to 15 THz, attributed to intraband absorption by excess free electrons in few-layer MoS_2_, WSe_2_, and their heterostructures. Furthermore, based on low-wavenumber Raman spectroscopy results, we propose a THz photon-to-phonon transition mechanism. When the few-layer MoS_2_ and WSe_2_ in the 2D heterostructure absorb photons with energies that are integer multiples of their own phonon energies, they release phonons corresponding to those energy multiples. These newly released phonons participate in phonon scattering, leading to thermal dissipation and subsequently causing an increase in the temperature of the heterostructure. Such a mechanism is corroborated by theoretical calculations of the phonon spectrum of MoS_2_ and WSe_2_. Additionally, by establishing a thermal conduction model for the heterostructure, we systematically investigated the thermodynamic changes in the heterostructure under continuous wave irradiation from 0.5 to 15 THz. The simulation results indicate that the overall temperature of the heterostructure increases and reveal the presence of the Seebeck effect arising from the temperature gradient.

## 2. Experiments and Methods

### 2.1. MoS_2_/WSe_2_/MoS_2_ Heterosruacture Fabrication

First, the bottom n-type MoS_2_ few layers were mechanically exfoliated using 3M adhesive tape on the (300 nm/500 μm) SiO_2_/Si substrates. Then, with the assistance of polydimethylsiloxane (PDMS) for transfer, the p-type WSe_2_ and another n-type MoS_2_ few-layers were exfoliated and sequentially transferred onto the bottom MoS_2_ flake using optical micromanipulator alignment technology. To avoid a short circuit, the top MoS_2_ layer should not come into direct contact with the bottom MoS_2_ layer.

### 2.2. Analytical Methods

The optical images were recorded for the fabrication of the vertically stacked vdW MoS_2_/WSe_2_/MoS_2_ heterostructure using an optical microscope (Olympus: BX51, Tokyo, Japan) equipped with a 50× objective lens (N.A. = 0.50) and a quartz-tungsten-halogen lamp (100 W), operating in reflection mode.

Raman and PL spectra were obtained at room temperature using a Raman spectrometer (Renishaw: InVia Reflex, Wotton-under-Edge, UK) equipped with a CCD detector and a 50× objective (Leica, Wetzler, Germany, model 11566074, N.A. = 0.75). The measurements were performed in a backscattering configuration. The laser used was a 532 nm solid-state laser (second harmonic from an Nd:YAG laser, model: RL532C, Renishaw), with a power of 52 mW. The laser was vertically incident on the sample surface. Both Raman and PL signals were then collected from the same side through reflection. The spectrometer was equipped with a diffraction grating of 2400 l/mm, providing high spectral resolution. The spectrometer covered a broad spectral range from 5 cm^−1^ to 3200 cm^−1^, ensuring the detection of both low- and high-frequency Raman modes. Rayleigh scattering was effectively suppressed using a Renishaw ultra-narrow-band notch filter (supplied by Renishaw, model: VBG) integrated into the spectrometer system, which facilitated the reliable acquisition of low-wavenumber Raman spectra.

The peak selection criteria were based on signal-to-noise (SNR) analysis:

(1) $$\mathrm{SNR}=\frac{I_{\mathrm{peak}}-I_{\mathrm{baseline}}}{\sigma_{\mathrm{noise}}}$$

where *I*_peak_ represents the intensity of the low-wavenumber Raman peak, *I*_baseline_ is the baseline intensity, and *σ*_noise_ is the standard deviation of the noise background. In our study, when the SNR exceeded 3, we claimed that the signal was a real Raman mode rather than noise.

The atomic force microscope study (AFM) for structural and morphological measurements was conducted using a Veeco Dimension-Icon system scanning probe microscope (Bruker Inc.: Dimension fastscan bio, Billerica, MA, USA) with SiN probes (Scanasyst-Air, calibrated spring constants of 0.3–0.5 N m^−1^ and a nominal tip radius of 2–5 nm). Topographic characterization was carried out in Peak Force Tapping mode under ambient conditions.

Time-resolved THz spectroscopy characterizations were conducted using a fiber-coupled THz time-domain spectroscopy (THz-TDS) system (TeraFlash pro, Toptica, Pittsford, NY, USA). In order to analyze the THz absorption properties of the few-layer MoS_2_, WSe_2_, and their vertically stacked heterostructures prepared on a high-resistance Si substrate (500-μm thickness covered by a 300-nm SiO_2_ layer, *ρ*~20,000 Ω·cm), THz-TDS spectroscopy measurements were conducted at room temperature. The large-area MoS_2_ and WSe_2_ flakes were purchased from the manufacturer (Shenzhen SixCarbon Technology Co., Ltd., Shenzhen, China) and were grown using the CVD method. The large-area MoS_2_/WSe_2_/MoS_2_ heterostructure was prepared using a wet chemical etching process (the detailed method is shown in the Supporting Information Section S1). The experimental samples for the TDS measurements should be at least 1.5 cm × 1.5 cm in area. For a specific TDS measurement, the sample was attached to a hollow iron plate for testing, and the THz wave focused on the sample with a spot size of 3 mm. The test chamber was filled with high-purity N_2_ to ensure that the air humidity was less than 10%. The TDS spectrum for the sample was recorded using a time constant of 300 ms, an integration time of 900 ms, a step size of 1.5 µm for distance changes between the excitation and probe beams, and a scan point number of 700.

The density functional theory (DFT) calculations were performed using the Vienna Ab Initio Simulation package (VASP) [27,28]. Specifically, in order to unveil the origins of the phonons, we calculated the electronic structure and force constant matrices using DFT implemented in the VASP and phonopy [29,30] package. The DFT calculations used the generalized gradient approximation (GGA) [31] of Perdew–Burke–Ernzerhof (PBE) [32] as the exchange-correlation function, and the plane wave cutoff energy was set to 520 eV, and the k-point mesh was a Gamma-centered 6 × 6 × 1 Monkhorst–Pack grid. Rigorous convergence tests were conducted to determine the computational parameters, as we had previously done in calculations of the dielectric functions of vdW polar crystals [33]. Accordingly, the plane wave cutoff energy was set to 520 eV, and the *k*-point mesh was a Gamma-centered 6 × 6 × 1 Monkhorst–Pack grid. The energy convergence accuracy was set to 1 × 10^−10^ eV, while the force convergence criterion for each atom was set to 1 × 10^−8^ eV/Å. With these parameters, we calculated the Born effective charge tensor [34] using the DFT linear response scheme combined with the iterative Green’s function method of density functional perturbation theory and then obtained the phonon spectrum in a 3 × 3 × 2 supercell combined with the second-order force constants calculated using the phonopy package.

The finite-element method based on triangular prismatic elements was conducted using an in-house-developed solver. This method demonstrates a robust capability in addressing the multiscale characteristics inherent to geometric models. The transient heat conduction equation was solved via an implicit backward differentiation formula with a stabilized time-stepping strategy (Δ*t*: 0.001–1 s). Material properties include thermal conductivity, density, and specific heat capacity. Boundary conditions enforced natural convective heat transfer at the air and large-area silicon substrate interfaces (h = 5–25 W·m^−2^·K^−1^), with prescribed thermal source constraints additionally applied. Temperature-time curves at the heterostructure centers were extracted through linear interpolation in the course of post-processing.

## 3. Results and Discussion

In our study, we focused on the 2D vdW MoS_2_/WSe_2_/MoS_2_ heterostructure, which is uniquely suited for optoelectronic applications due to its synergistic material properties and interfacial advantages. Both materials are TMDs with complementary electronic characteristics: MoS_2_ provides high electron mobility (~200 cm^2^/V·s), while WSe_2_ exhibits superior hole mobility (>100 cm^2^/V·s), enabling balanced charge transport. Their bandgaps (1.2–1.9 eV for MoS_2_, 1.2–1.7 eV for WSe_2_) form a type-II staggered alignment, enhancing photocarrier separation and suppressing recombination—critical for photodetectors and THz applications. The type-II band alignment in MoS_2_/WSe_2_ outperforms alternatives like MoS_2_/WS_2_ (type-I) or MoSe_2_/WSe_2_ (type-I), which suffer from inefficient charge separation. Additionally, the small lattice mismatch (3.8%) minimizes interfacial strain and defects, ensuring high-quality heterostructures with efficient phonon transport. In contrast, other TMD pairs (e.g., MoS_2_/MoTe_2_, WSe_2_/WS_2_) exhibit larger mismatches (>4%), degrading device performance. Experimentally, MoS_2_ and WSe_2_ are air-stable and mechanically robust, enabling reliable exfoliation and precise stacking. This contrasts with air-sensitive materials like black phosphorus or ReS_2_, which complicate fabrication. The trilayer n-p-n configuration further enhances optoelectronic performance, facilitating strong in-plane p-n junctions and efficient carrier extraction. Crucially, the heterostructure exhibits strong THz photon-phonon coupling, as evidenced by low-wavenumber Raman modes and phonon simulations. THz irradiation excites interlayer vibrations, converting photon energy into lattice heating—a key mechanism for THz detection. This, combined with its thermal and electronic properties, makes MoS_2_/WSe_2_/MoS_2_ an optimal platform for advanced optoelectronics.

**Figure 1:**
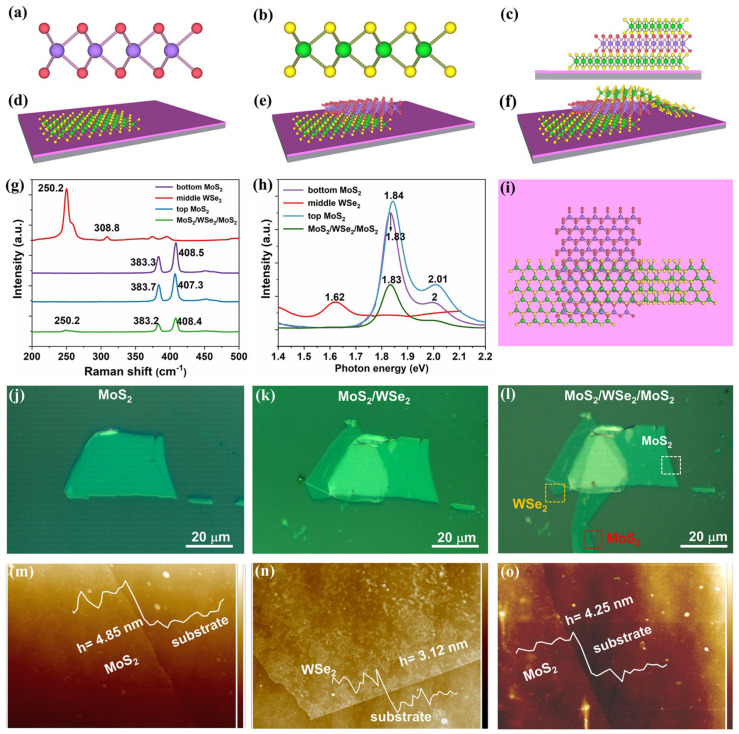
vdW MoS_2_/WSe_2_/MoS_2_ heterostructure illustrations and morphological characterizations. (**a**–**f**) Schematic illustration of heterostructure fabrication procedures. (**a**,**b**) The MoS_2_ and WSe_2_ space-cell structure model. (**c**) The cross−sectional view of the vertical MoS_2_/WSe_2_/MoS_2_ heterostructure. (**d**) The few−layer MoS_2_ on SiO_2_/Si substrate. (**e**) The vertical MoS_2_/WSe_2_ heterostructure. (**f**) The vertical MoS_2_/WSe_2_/MoS_2_ heterostructure. (**g**,**h**) Raman (**g**) and photoluminescence (PL) (**h**) spectra of the bottom MoS_2_ (purple line), middle WSe_2_ (red line), top MoS_2_ (blue line), and overlapping regions of the three flakes (green line). (**i**) The top view of the heterostructure. (**j**–**l**) The optical microscope images of the vertically stacked structure; scale bar: 20 μm. (**m**–**o**) AFM images of the three−box area shown in (**l**). Insets: the AFM height profiles of bottom MoS_2_ (4.85 nm), middle WSe_2_ (3.12 nm), and top MoS_2_ (4.25 nm), respectively.

The schematic illustration of the MoS_2_/WSe_2_/MoS_2_ heterostructures is depicted in Figure 1a–f,i. The space-cell structure model of MoS_2_ and WSe_2_ is shown in Figure 1a,b. The diagrams of the preparation processes of the heterostructure are displayed in Figure 1d–f. The cross-sectional view and top view of the vertical MoS_2_/WSe_2_/MoS_2_ heterostructure are shown in Figure 1c,i. The corresponding optical microscope images captured after each transfer procedure during the whole fabrication procedure are given in Figure 1j–l. The Raman spectra of the MoS_2_, WSe_2_, MoS_2_/WSe_2_, and MoS_2_/WSe_2_/MoS_2_ heterostructures are presented in Figure 1g. Distinct Raman peaks are observed at ~383.3 cm^−1^ and 408.5 cm^−1^, corresponding to the vibration modes $E_{2g}^{1}$ (in-plane) and A_1g_ (out-of-plane) of MoS_2_, respectively. The wavenumber difference Δd of these two vibration modes can simply reflect the layer number of the MoS_2_ [35]. The Δd values of the bottom and top MoS_2_ are 25.2 cm^−1^ and 23.6 cm^−1^, respectively. Both values suggest more than five layers of the two MoS_2_ layers. As for the Raman peaks of WSe_2_, a strong peak at 250.2 cm^−1^ is related to the A_1g_ mode, and a small signature at 306.8 cm^−1^ may be assigned to the normally inactive B_2g_ mode. Notably, the Raman peaks of MoS_2_ in the overlapped region exhibit slight broadening and shifting, indicating charge transfer across the interfaces. Consequently, a depletion layer was formed at the MoS_2_/WSe_2_/MoS_2_ vdW heterostructure [36].

Figure 1h depicts the photoluminescence (PL) spectra of the vertically stacked heterostructure. Two peaks of the top MoS_2_ layer are observed at 1.84 eV (673.9 nm) and 2.0 eV (620 nm), respectively. They correspond to the A_1_ and B_1_ direct excitonic transitions with the energy splitting due to valence band spin-orbital coupling [37]. The A_1_ and B_1_ peaks of the bottom MoS_2_ layers appear to be slightly shifted to 1.83 eV (677.6 nm) and 1.99 eV (623.1 nm), respectively. This suggests that the bottom and top MoS_2_ layers have different layer numbers. The middle WSe_2_ layer exhibits a PL peak at 1.62 eV (765.4 nm), corresponding to its indirect band gap [38]. However, the PL intensity is strongly suppressed in the overlapped region of the three layers (Figure 1h, green line), which can be attributed to the radiative recombination of the spatially separated electron-hole pairs in different layers.

AFM images recorded at three different regions (marked with colored boxes in Figure 1l) are shown in Figure 1m–o. They clearly display the atomically flat surfaces of the mechanically exfoliated MoS_2_ and WSe_2_ layers. AFM height profiles extracted from each layer of MoS_2_/WSe_2_/MoS_2_ are shown in the insets. The results display the heights of the three layers, i.e., 4.85 nm, 3.12 nm, and 4.25 nm, respectively, for the bottom MoS_2_, middle WSe_2_, and top MoS_2_. These thicknesses are consistent with the Raman results shown in Figure 1g. AFM results demonstrate that the atoms within the layers are uniformly arranged, and the interfaces of the stacked structure are flat without any dislocations or defects, possessing a high-quality atomic-level crystalline interface [39].

**Figure 2:**
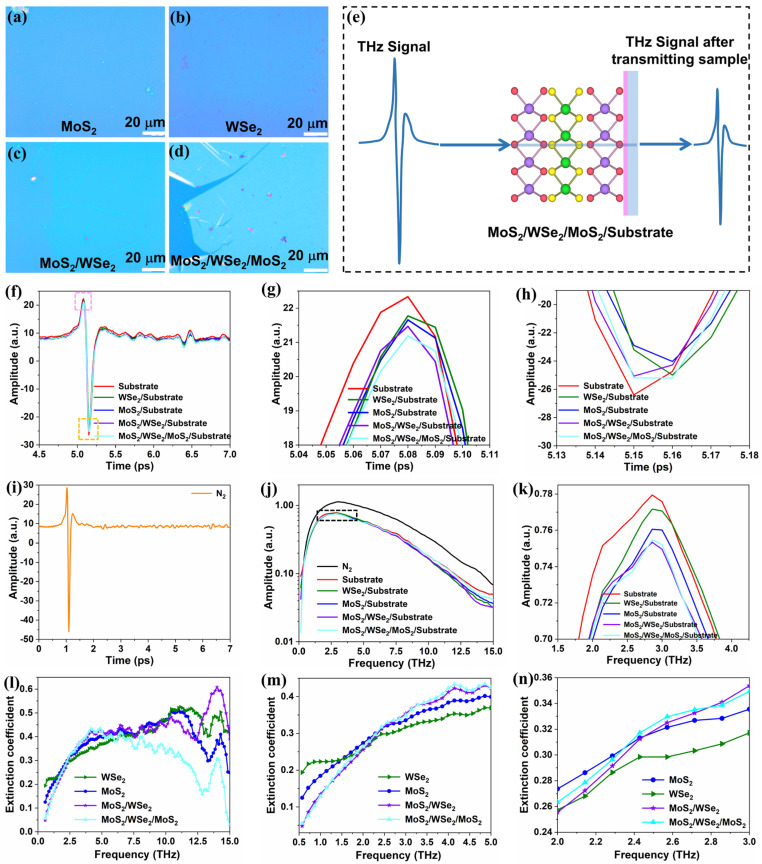
THz time−domain spectral characterization of large−area 2D vdW MoS_2_, WSe_2_ crystals and their heterostructures. (**a**–**d**) Optical micrographs of large−area MoS_2_, WSe_2_, MoS_2_/WSe_2_, and MoS_2_/WSe_2_/MoS_2_, respectively; scale bar: 20 μm. (**e**) Schematic illustration of the THz−TDS system used for the experiment. (**f**) Time−domain spectra for blank substrate, MoS_2_ on the substrate, WSe_2_ on the substrate, MoS_2_/WSe_2_ on the substrate, and MoS_2_/WSe_2_/MoS_2_ on the substrate. (**g**–**h**) Enlarged spectra of the pink and orange dotted boxes in (**f**). (**i**) Time−domain spectrum of the N_2_ atmosphere. (**j**) Frequency−domain spectra obtained by Fourier transform of the time−domain spectra of N_2_ atmosphere, blank substrate, MoS_2_, WSe_2_, MoS_2_/WSe_2_, and MoS_2_/WSe_2_/MoS_2_. The black box: the amplitude variations of different materials at frequencies ranging from 1.5 to 4.5 THz. (**k**) Enlarged spectra of the black dotted boxes in (**j**). (**l**–**n**) The calculated extinction coefficient curves of the frequency−domain spectra of MoS_2_, WSe_2_, MoS_2_/WSe_2_, and MoS_2_/WSe_2_/MoS_2_: (**l**) 0.5–15 THz, (**m**) 0.5–10 THz, (**n**) 0.5–5 THz.

Figure 2a–d depicts the optical micrographs of large-area MoS_2_, WSe_2_, MoS_2_/WSe_2_, and MoS_2_/WSe_2_/MoS_2_, respectively. A schematic illustration of the TDS spectroscopy is shown in Figure 2e. Figure 2f displays THz-TDS results from the blank substrate, MoS_2_, WSe_2_, MoS_2_/WSe_2_, and MoS_2_/WSe_2_/MoS_2_. The time-domain peak and valley of the THz wave transmitting through large-area vdW MoS_2_, WSe_2_, and their heterostructures on high-resistance Si are ~5 ps. Figure 2i displays THz-TDS results from the N_2_ atmosphere. The time-domain peak position of the THz signal transmitting through N_2_ absorption is ~1 ps.

Figure 2g,h presents a detailed analysis of the time-domain spectra (shown around the 5 ps region in Figure 2f) for different 2D vdW MoS_2_, WSe_2_, and their heterostructures. The strength of THz absorption is represented by the difference between the adjacent peaks (or valleys) of the time-domain signal. As the THz signal passes through WSe_2_, MoS_2_, MoS_2_/WSe_2_, and MoS_2_/WSe_2_/MoS_2_, the signal intensity gradually decreases. The THz electric field transmitting through the blank substrate, MoS_2_/substrate, WSe_2_/substrate, MoS_2_/WSe_2_/substrate, and MoS_2_/WSe_2_/MoS_2_/substrate is defined as *E*_sub_(*τ*), *E*_M_(*τ*), *E*_W_(*τ*), *E*_M/W_(*τ*), and *E*_M/W/M_(*τ*), respectively. The maximum values of *E*_M_(*τ*), *E*_W_(*τ*), *E*_M/W_(*τ*), and *E*_M/W/M_(*τ*) respectively show reductions of 3.03%, 2.5%, 3.9%, and 5.1% compared to *E*_sub_(*τ*). This attenuation of the THz transmission arises from absorption in the few-layer MoS_2_ and WSe_2_, as well as their heterostructures, either due to the intraband transition of excess free electrons [40] or phonon excitation, as discussed below.

The frequency-domain spectrum is obtained by conducting a Fourier transform of the TDS spectrum. In the range of 0.5–20 THz, clear spectral lines in the frequency domain can be observed, as shown in Figure 2j. The enlarged frequency domain spectrum from 1.5 to 4.5 THz (regions marked by blue dotted boxes in Figure 2j) is presented in Figure 2k. The THz wave amplitude in the frequency domain exhibits a gradual decrease across the material sequence: WSe_2_, MoS_2_, and MoS_2_/WSe_2_ to MoS_2_/WSe_2_/MoS_2_.

The transmittance coefficient of the material can be calculated according to [41]:

(2) $$\mathrm{Transmittance}=\frac{E_{\mathrm{sample}}{\left(\tau \right)}^{2}}{E_{\mathrm{substrate}}{\left(\tau \right)}^{2}}$$

where *E*_sample_ (*τ*) is the amplitude of the sample signal, and *E*_substrate_ (*τ*) is the amplitude of the substrate signal. The extinction coefficient of the material can be calculated according to

(3) Extinction coefficient=1−Transmittance

The extinction coefficient varies across the frequency ranges of 0.5–15 THz, 0.5–10 THz, and 0.5–5 THz, as shown in Figure 2l–n. In the THz frequency range of 0.5–2 THz, the extinction coefficient of few-layer MoS_2_ and WSe_2_ is higher than that of the heterostructure. In the range of 2–5 THz, the extinction of THz signals of MoS_2_/WSe_2_ and MoS_2_/WSe_2_/MoS_2_ heterostructures gradually increases, surpassing the values observed in few-layer MoS_2_ and WSe_2_. Significant signal fluctuations emerge for the frequency range above 5 THz. In this spectral range, the extinction coefficient of few-layer MoS_2_, WSe_2_, and MoS_2_/WSe_2_ increases gradually, whereas that of MoS_2_/WSe_2_/MoS_2_ declines. According to the data in Figure 2n, the extinction coefficient of WSe_2_, MoS_2_, MoS_2_/WSe_2_, and MoS_2_/WSe_2_/MoS_2_ was 30%, 31.8%, 32.1%, and 32.6% at 2.52 THz.

In the traditional photon detection mechanism, the photon energy must exceed the semiconductor band gap energy to enable electron transitions from the valence band to the conduction band, thereby altering the conductivity [42]. However, the THz band from 0.1 to 10 THz corresponds to a photon energy of 0.414 to 41.4 meV, which is much smaller than the band gap of MoS_2_ (1.83 eV) and WSe_2_ (1.62 eV). The absorption of the THz wave in the vdW crystals and heterostructures may, therefore, arise from phonon excitations by the THz wave. For THz waves with frequencies less than 1 THz, the corresponding wavenumber (the reciprocal of the wavelength) is less than 33 cm^−1^. This suggests that it is possible to explore the coupling between THz photons and phonons in TMDs and their heterostructures using low-wavenumber Raman spectroscopy, which can reveal the phonon modes in the range of 0–100 cm^−1^.

**Figure 3:**
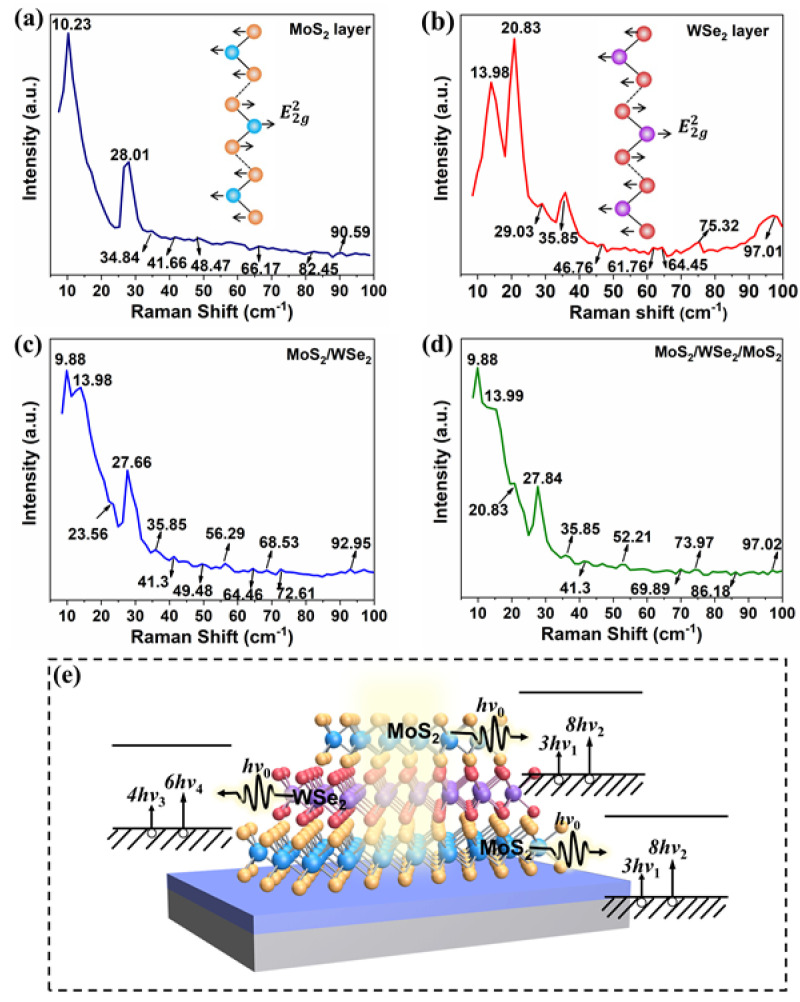
The low-wavenumber Raman analysis for vdW MoS_2_ and WSe_2_ and their heterostructures. (**a**–**d**) Low−wavenumber Raman spectra of MoS_2_ (**a**), WSe_2_ (**b**), MoS_2_/WSe_2_ (**c**), and MoS_2_/WSe_2_/MoS_2_ (**d**), respectively. The atomic vibrations shown in (**a**,**b**) correspond to $E_{2g}^{2}$ modes, which are associated with the Raman peaks at 10.23 cm^−1^/0.31 THz (**a**) and 13.98 cm^−1^/0.42 THz (**b**), respectively. (**e**) Schematic illustration of the THz photon–phonon transition mechanism.

A 532-nm laser is used to obtain the low-wavenumber Raman spectra of MoS_2_, WSe_2_, MoS_2_/WSe_2,_ and MoS_2_/WSe_2_/MoS_2_. According to the SNR of the spectral bumps, different Raman peaks can be assigned (Tables S1 and S2 in the Supporting Information). Figure 3a shows the Raman spectra of a few layers of MoS_2_. Two peaks at 10.23 cm^−1^ and 28.01 cm^−1^ can be observed, which respectively correspond to frequencies of 0.31 THz and 0.84 THz. Figure 3b presents spectra obtained from few-layer WSe_2_. Peaks at 13.98 cm^−1^, 20.83 cm^−1^, 29.03 cm^−1^, 35.85 cm^−1^, 61.9 cm^−1^, 75.32 cm^−1^, and 97.01 cm^−1^, corresponding to frequencies of 0.42 THz, 0.64 THz, 0.87 THz, 1.08 THz, 1.86 THz, 2.26 THz, and 2.91 THz, respectively, can be recorded. For the MoS_2_/WSe_2_ heterostructure, five peaks, located at 9.88 cm^−1^ (0.3 THz), 13.98 cm^−1^ (0.42 THz), 23.56 cm^−1^ (0.71 THz), 27.66 cm^−1^ (0.83 THz), and 35.85 cm^−1^ (1.08 THz), can be recorded (Figure 3c). Figure 3d depicts the low-wavenumber Raman spectra of the MoS_2_/WSe_2_/MoS_2_ heterostructure, with peaks at 9.88 cm^−1^, 13.99 cm^−1^, 20.83 cm^−1^, and 27.84 cm^−1^, corresponding to frequencies of 0.3 THz, 0.42 THz, 0.63 THz, and 0.84 THz, respectively. Table 1 provides a summary of the above results.

Based on the results of the above low-wavenumber Raman spectroscopy, we discover several typical phonon modes of MoS_2_ and WSe_2_, corresponding to the following frequencies:

MoS_2_: 10.23 cm^−1^ to 0.31 THz (*ν*_1_), 28.01 cm^−1^ to 0.84 THz (*ν*_2_).

WSe_2_: 13.98 cm^−1^ to 0.42 THz (*ν*_3_), 20.83 cm^−1^ to 0.64 THz (*ν*_4_).

For a 2.52-THz photon, these frequencies correspond respectively to ~8 (*ν*_1_), 3 (*ν*_2_), 6 (*ν*_3_), and ~4 (*ν*_4_). According to these observations, we propose a mechanism for THz absorption in the vdW crystals and heterostructures. Specifically, when a THz wave irradiates the vdW crystals, the vdW lattices will absorb the photons by emitting corresponding optical phonons, provided the photon energy equals an integer multiple of the optical phonon energies in the crystals. Based on this mechanism and the phonon energies extracted from the low-wavenumber Raman spectroscopy measurements, we assume that the TMDs used in our study may strongly absorb THz photons with energies that are integer multiples of *ν*_1_ to *ν*_4_. Figure 3e illustrates this photon-to-phonon transition process in our heterostructure. Specifically, when the MoS_2_ lattice absorbs THz photons at 2.52 THz, eight phonons (*ν*_1_) or three phonons (*ν*_2_) can be released. Similarly, when the WSe_2_ lattice absorbs a 2.52-THz photon, six phonons (*ν*_3_) and four phonons (*ν*_4_) can be released. These newly released phonons enhance the lattice vibration, thus leading to an increment in the crystal temperature. This is the main mechanism governing the THz wave absorption in our 2D MoS_2_/WSe_2_/MoS_2_ heterostructures. It should be noted that bulk MoS_2_ and WSe_2_ are centrosymmetric crystals in principle. In such systems, Raman-active phonon modes are infrared-inactive, meaning they cannot be directly excited by THz photons. However, the samples in our experiment consist of few-layer structures. When the layer number is odd, the crystal loses centrosymmetry, rendering most phonon modes both Raman- and infrared-active. In our study, the layer numbers are 5 for the top MoS_2_ layer, 7 for the bottom MoS_2_ layer, and 5 for the middle WSe_2_ layer. Consequently, THz photon absorption in these samples is still governed by the aforementioned multi-phonon excitation processes.

**Figure 4:**
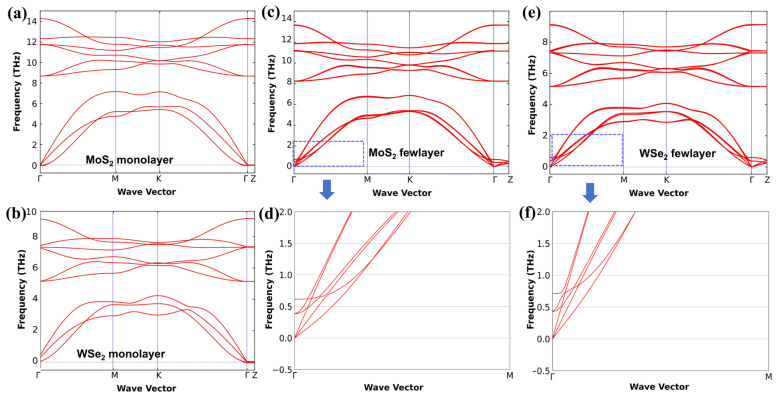
The phonon dispersion analysis for the monolayer and bulk MoS_2_ and WSe_2_ (theoretical results). (**a**) Monolayer MoS_2_. (**b**) Monolayer WSe_2_. (**c**) Bulk MoS_2_. The purple dashed box: the calculation results of the phonon dispersion at the Γ–M point. (**d**) Amplifying the phonon dispersion of bulk MoS_2_ from (**c**) in the blue-dashed box at the Γ–M point. (**e**) Bulk WSe_2_. The purple dashed box: the calculation results of the phonon dispersion at the Γ–M point. (**f**) Enlarged phonon dispersion of bulk WSe_2_ from (**e**) in the blue−dashed box at the Γ–M point.

Through theoretical calculations, we plotted the phonon dispersion of monolayer and bulk MoS_2_ and WSe_2_ in Figure 4. Combined with Tables S3 and S4 in the Supporting Information, the corresponding phonon frequencies of MoS_2_ and WSe_2_ with different thicknesses at the Γ points are shown. There are three acoustic branches, whose frequencies are zero at the Γ point in the phonon dispersion of each crystal. In particular, for the bulk crystals, three branches are close to the acoustic ones in the dispersion curves. The vibrations of atoms in each of these three branches are in phase within the same layer but are oppositely phased in adjacent layers. These are new optical phonons that are absent in monolayer crystals.

The values of the new optical phonon branches in bulk MoS_2_ (Figure 4c,d) at the Γ point are about 0.42 THz and 0.71 THz, respectively. These agree with the results for low-wavenumber Raman experiments of MoS_2_ (0.31 THz and 0.84 THz). The values of the new optical phonon branches in bulk WSe_2_ at the Γ point are 0.38 THz and 0.61 THz, respectively (Figure 4e,f). These also agree with the results for low-wavenumber Raman experiments of WSe_2_ (0.42 THz and 0.64 THz).

Based on the previous TDS results, few-layer MoS_2_, WSe_2_, and their heterostructures all have strong absorption of THz waves. After absorption, they will release phonons and lead to an increase in crystalline temperature. We then simulate the thermal responses of different vdW crystals, accounting for heat dissipation from the large-area silicon substrate and air. We employ an in-house thermal simulation solver, utilizing a finite element method based on triangular prismatic elements to establish a thermal conduction model for the vdW MoS_2_/WSe_2_/MoS_2_ heterostructures. The THz frequency range used in our simulations was 0.5–15 THz, and the physical parameters of the vdW heterostructure were derived from experimentally prepared samples, as shown in Figure S1 and Table S5. A series of simulation results related to the temperature changes in the vdW heterostructure is then readily obtained.

**Figure 5:**
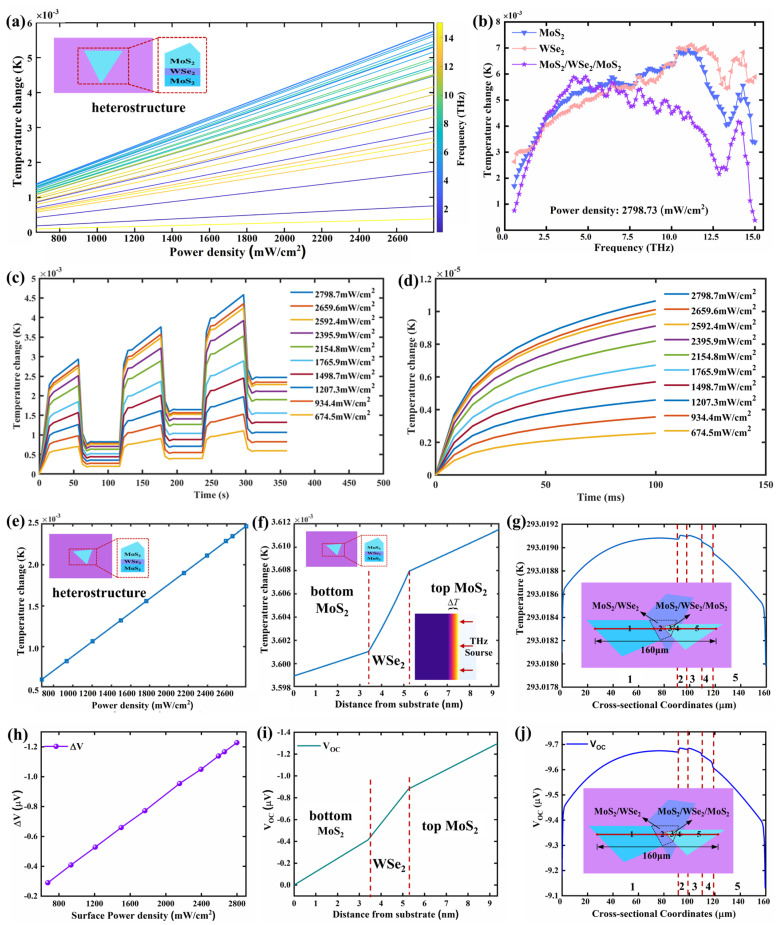
Simulations of thermal responses under THz irradiation of the MoS_2_/WSe_2_/MoS_2_ heterostructure. (**a**) Temperature changes, Δ*T*, of the heterostructure upon irradiation by THz waves of 0.5–15 THz with varied power densities. (**b**) Temperature changes of MoS_2_, WSe_2_, and the MoS_2_/WSe_2_/MoS_2_ heterostructure as a function of the irradiation frequency. The power density is kept at 2798.73 mW/cm^2^. (**c**) Time−resolved temperature changes of the MoS_2_/WSe_2_/MoS_2_ heterostructure upon irradiation with different THz power densities. The THz wave frequency is kept at 2.52 THz. (**d**) The temperature rise of the heterostructure over time within 100 ms of THz radiation under different power densities. (**e**) The temperature increases Δ*T* of the heterostructure as a function of the THz power density. (**f**) The variation of Δ*T* of each layer in the heterostructure along the direction perpendicular to the sample surface. (**g**) The temperature distribution along the red solid line. (**h**) The thermal voltage, Δ*V*, across the heterostructure as a function of the THz power density. (**i**) The open-circuit voltage *V*_OC_ of each layer in the heterostructure along the direction perpendicular to the sample surface. (**j**) The variation of *V*_OC_ along the red solid line.

First, we investigated the temperature changes, ∆*T*, in the stacked region of the heterostructure upon THz irradiation from 0.5 THz to 15 THz at different power densities. The irradiation duration, Δ*t*, is 120 s, consisting of 60-s radiation intervals alternating with 60-s recovery periods. As shown in Figure 5a, the heterostructure exhibits a temperature rise after irradiation in the 0.5–15 THz frequency range. In particular, at the same power density, the ∆*T* increases against the frequency for 0.5 to 5 THz, then decreases as the irradiation frequency is reduced. According to the aforementioned TDS results, the extinction ratio of the heterostructure shows an increasing trend within the 0.5–5 THz range, while it gradually decreases above 5 THz. This frequency-dependent temperature rise, therefore, strongly correlates with the extinction coefficient trends observed in the TDS extinction. In the range of the 0.5–15 THz frequency band, we calculated the Δ*T* in the bottom MoS_2_ layer, the middle WSe_2_ layer, and the top MoS_2_ layer under continuous irradiation by three THz pulses with different power densities. The results are shown in Figures S2–S4.

Additionally, we compared the temperature changes Δ*T* of individual MoS_2_, WSe_2_, and the MoS_2_/WSe_2_/MoS_2_ heterostructure irradiated by different THz frequencies at a surface power density of 2798.73 mW/cm^2^, as presented in Figure 5b. The results reveal that within the ranges of 0.5–2.5 THz and 5–15 THz, the temperature rise of individual MoS_2_ and WSe_2_ is greater than that of the heterostructure. However, within the range of **2.5–5 THz,** the trend is reversed, with the heterostructure exhibiting a higher temperature increase compared to the individual materials. More attention should be given to the frequency ranges below 2.5 THz and above 10 THz. In the frequency range below 2.5 THz, the thermal simulation results indicate that Δ*T* in the heterojunction is significantly influenced by interlayer thermal conductivity differences. The high thermal conductivity of the top MoS_2_ layer (100 W/(m·K)) accelerates heat diffusion to the substrate (SiO_2_/Si), while the low thermal conductivity of the middle WSe_2_ layer (40 W/(m·K)) partially hinders heat transfer. However, the overall thermal resistance distribution remains dominated by rapid lateral heat diffusion. The competition between these heat diffusion pathways results in a significant reduction in the temperature gradient within the heterojunction at lower frequencies, with the temperature rise being lower than that of monolayer materials (Figure 5b). These results predict that, in low-frequency applications, the thermal response of the heterojunction is likely more dependent on the thermal dissipation capacity of the substrate rather than interlayer cooperative effects. In the frequency range above 10 THz, the simulation results suggest that the thermal response of the heterojunction is likely limited by the thermal diffusion rate at the surface and interlayer thermal conduction, rather than the heat generation capability of the material. The reduced absorption depth of high-frequency THz energy in the heterojunction likely causes heat to become more concentrated in the top MoS_2_ layer, while the high thermal conductivity of the top layer further accelerates heat dissipation to the environment (natural convection coefficient *h* = 5−25 W·m^−2^·K^−1^). Furthermore, the heat distribution at high frequencies is likely more significantly affected by interlayer thermal conduction. Due to the thermal conductivity differences between the layers, heat transfer between layers involves scattering and uneven energy distribution. These factors make the conduction path of heat within the material more complex, increasing energy dissipation and limiting the accumulation of overall temperature rise.

We then simulated the time-resolved temperature rise profiles of the heterostructure under THz irradiation with different power densities, as shown in Figure 5c. It can be observed that following three consecutive THz pulses at 2.52 THz, the heterostructure exhibits nonlinear thermal responses. Specifically, during each pulse cycle, the temperature rises rapidly before transitioning to a sublinear growth phase without saturation. Upon pulse termination, an initial rapid cooling phase is followed by gradual thermal equilibration. Simultaneously, within a single pulse cycle, the heat of the heterostructure accumulated during the first 60 s of irradiation is mostly dissipated through thermal diffusion and convection in the subsequent 60 s. However, after continuous irradiation by three THz pulse cycles, the heterostructure still retains a portion of heat accumulation, manifesting as a temperature increase background. Under the maximum power density of 2798.7 mW/cm^2^, the final temperature rise of the heterostructure is approximately 2.5 mK.

To better understand the rapid temperature change process, we extracted the temperature rise curves of the heterostructure within the first 100 ms of THz radiation under different power densities from Figure 5c and plotted them in Figure 5d. Clearly, the Δ*T* of the heterostructure exhibits a nonlinear increase within the first 50 ms of irradiation at 2.52 THz waves. Beyond 50 ms, the Δ*T* demonstrates a linear growth trend over time, and the slope of the temperature curve shows a positive correlation with the power density of the THz wave irradiation on the sample.

We continue to investigate the Δ*T* in the stacked region of the heterostructure under continuous irradiation at 2.52 THz by three THz pulses of varying power densities. The results are displayed in Figure 5e. The temperature of the stacked structure region of the heterostructure increased overall after being irradiated by three consecutive THz pulses. Moreover, the Δ*T* increases linearly against the power density. This observation indicates that as the THz power density increases, the energy radiated onto the surface of the stacked structure per unit time also increases. Consequently, the material absorbs more THz photons, which are then converted into additional lattice thermal energy, leading to a rise in the material temperature. These findings indicate that the THz response mechanism of the heterostructure can be attributed to the photothermal effect [43]. Additionally, we also calculated the temperature changes in each layer of the heterostructure after THz irradiation. The simulation results are shown in Figure S5. The results indicate that the bottom MoS_2_ layer, the middle WSe_2_ layer, and the top MoS_2_ layer of the heterostructure all exhibited similar trends of temperature rise.

Photothermoelectric effect (PTE) describes photon-to-electron conversion due to the concentration or temperature gradient of photogenerated hot carriers in materials under light excitation. The gradient can drive the directional flow of electrons, thereby generating open-circuit-voltage or short-circuit-current photoelectric responses [44]. In the case of our 2D vdW heterostructures, after the crystalline lattice absorbs THz photons, the localized temperature rises within the material, forming a temperature gradient. According to the Seebeck effect [45], the temperature difference between the two terminals of the material can generate a potential difference (thermal voltage) as follows:

(4) ΔV=S×ΔT

where S denotes the Seebeck coefficient and Δ*T* represents the temperature gradient. For the vdW MoS_2_/WSe_2_/MoS_2_ heterostructure, the total Seebeck coefficient S_M/W/M_ can be calculated by the following formula [46]:

(5) $$S_{M/W/M}=\frac{\sigma_{\mathrm{BM}}{\times S}_{\mathrm{BM}}+\sigma_{\mathrm{MW}}\times S_{\mathrm{MW}}+\sigma_{\mathrm{TM}}\times S_{\mathrm{TM}}}{\sigma_{\mathrm{BM}}+\sigma_{\mathrm{MW}}+\sigma_{\mathrm{TM}}}$$

where *σ* is the conductivity of the material. The subscript “M/W/W” represents the MoS_2_/WSe_2_/MoS_2_, the subscript “BM” represents bottom MoS_2_, the subscript “MW” represents middle WSe_2_, and the subscript “TM” represents top MoS_2_. According to the relevant literature on the thermoelectric effect of MoS_2_ and WSe_2_ of different thicknesses [47,48], the Seebeck coefficient S_BM_ and the conductivity *σ*_BM_ of bottom MoS_2_ are ~−507 μV/W, 1670 S/m, respectively. The S_W_ and *σ*_W_ of middle WSe_2_ in the middle are about 50 μV/W and 34.5 S/m, respectively. For the top MoS_2_, the S_TM_ and *σ*_TM_ are approximately −494 μV/W and 1120 S/m, respectively. Combined with Equation (4), the total Seebeck coefficient S_M/W/M_ is calculated as −495.04 μV/W. Based on the variation of Δ*T* in heterostructure irradiated by different THz power densities (Figure 5e), the electric potential difference Δ*V* of the overall heterostructure can be calculated as a function of the THz power densities, as shown in Figure 5h. The Δ*V* of the heterostructure is linearly dependent on the THz power density, with a maximum value of 1.2 μV at the highest irradiation intensity.

Next, we explored the Δ*T* in each layer of the heterostructure along the direction perpendicular to the sample surface, as depicted in Figure 5f. From the bottom layer to the top layer, the temperature of each material gradually rises with distance from the silicon substrate. The bottom MoS_2_ layer, closest to the silicon substrate, exhibits the smallest temperature change after THz pulse irradiation. Although the middle WSe_2_ layer is relatively thin (with a thickness of 1.86 nm), the temperature difference between its upper and lower surfaces becomes the largest under the same conditions due to its lower thermal conductivity (40 W/(m·K)) compared to MoS_2_ (100 W/(m·K)).

The Seebeck coefficient in the vertical (out-of-plane) direction of the material shows distinct characteristics compared to that in the in-plane direction. According to the results reported in the relevant literature [49,50], the out-of-plane Seebeck coefficient of the MoS_2_ and WSe_2_ are approximately −115 μV/W and +129 μV/W, respectively. According to Equation (4) and Figure 5f, we calculated the open-circuit voltage *V*_OC_ distribution of each layer of the heterostructure in the direction perpendicular to the sample surface, as presented in Figure 5i. It can be seen that the *V*_OC_ distribution of each layer in the heterostructure is consistent with the temperature change of each layer. The above results further confirm the existence of the PTE effect.

Finally, we also explored the temperature distribution across the lateral region of the whole heterostructure surface, with the results presented in Figure 5g. From one end of the sample to the other, the temperature gradually increases from the region of the bottom MoS_2_ layer, reaching its maximum in the stacked region of the three crystals, and then gradually decreases in the region of the top MoS_2_ layer on the right side. Based on the temperature gradient across the lateral region in the whole heterostructure, we calculated the *V*_OC_ distribution using Equations (4) and (5). The results indicate that the *V*_OC_ distribution exhibits spatial consistency with the temperature variation profile, thereby providing further evidence for the PTE effect observed.

It is noted that all of the above simulations are conducted under room temperature (~25 °C) and low humidity (<10%) conditions, without directly investigating the effects of temperature and humidity variations on the aforementioned processes. Based on the current findings, temperature and humidity may regulate the photo-thermal-electric effect of the heterostructure through several mechanisms. First, our thermal simulations assumed fixed thermal conductivities (MoS_2_: 100 W/(m·K); WSe_2_: 40 W/(m·K)), but practical scenarios suggest thermal conductivity (*κ*) decreases with rising temperature. For example, $\kappa_{{\mathrm{MoS}}_{2}}$ may decline below 80 W/(m·K) at temperatures exceeding 100 °C. Such reductions would elevate thermal resistance and amplify the temperature gradient within the heterostructure. While simulations indicate a linear dependence of Δ*T* on THz power density (Figure 5e in our original manuscript), the temperature dependence of thermal conductivity *κ*(*T*) at elevated temperatures may disrupt this linear relationship. This deviation would arise primarily from reduced thermal diffusion rates, particularly at multilayer interfaces. Furthermore, the temperature-sensitive thermal diffusion properties of the SiO_2_/Si substrate may also disrupt overall thermal equilibrium [51]. Secondly, the Seebeck voltage (Δ*V* = S × Δ*T*) is influenced by the temperature dependence of the Seebeck coefficients in individual layers. If the Seebeck coefficients vary with temperature, the observed linear relationship between Δ*V* and power density (Figure 5h in our original manuscript) may deviate. Thirdly, under high humidity, adsorbed water films at heterojunction interfaces may form low*-κ* layers, exacerbating temperature gradients and impairing thermal conduction. Humidity fluctuations could also modulate material surface states and interfacial bonding, potentially altering Seebeck coefficients and their thermal response. Although current experiments are restricted to low humidity (<10%), future studies will systematically probe the role of humidity in thermo-electrical conversion. These issues will be explored systematically in our future study.

## 4. Conclusions

In the current study, we employed optical micromechanical arm alignment technology to successfully fabricate 2D vdW MoS_2_/WSe_2_/MoS_2_ few-layer structures and their heterostructures with a high-quality crystalline interface. THz-TDS measurements show obvious absorption in the heterostructure in the range of 0.5–15 THz. The few-layer WSe_2_, MoS_2_, MoS_2_/WSe_2_, and MoS_2_/WSe_2_/MoS_2_ exhibit an extinction coefficient of about 30%, 31.8%, 32.1%, and 32.6% at 2.52 THz, respectively. Several strong phonon modes are observed in the low-wavenumber Raman spectra of few-layer MoS_2_ and WSe_2,_ corresponding to frequencies of 0.31 THz and 0.84 THz for MoS_2_, and 0.64 THz and 0.42 THz for WSe_2_. The energy of an incident THz photon may be equal to that of a few such phonons. Therefore, we propose a THz photo-to-phonon transition mechanism. In the present heterostructure, absorption of THz photons by the few-layer MoS_2_ and WSe_2_ lattices results in the emission of an integer number of their respective optical phonons. These newly emitted phonons lead to the rise of the temperature of the heterostructure.

Theoretical simulations of the phonon dispersion show degenerate states in the acoustic phonon branches of monolayer MoS_2_ and WSe_2_, with these states lifting in few-layer structures. This further validates our proposed THz photon-to-phonon transition mechanism.

The thermal response simulation results show the overall temperature increase of the heterostructure subjected to the THz pulses. Additionally, the temperature change Δ*T* and the PTE voltage exhibit a linear increase with the THz power density. Because photon-phonon couplings are ubiquitous and important in THz photonics, the results of this work are expected to gain more applications in 6G communication, terahertz imaging, macromolecular detection, astronomy, etc.

## Figures and Tables

**Table 1:** The characteristics of low−wavenumber Raman modes and corresponding wavelengths, frequencies, and phonon energies of different TMDs and their heterostructures.

Materials	Raman Shift (cm^−1^)	Wavelength (cm)	Frequency (THz)	Phonon Energy (meV)
MoS_2_	10.23	0.097	0.31	1.28
	28.01	0.036	0.84	3.48
WSe_2_	13.98	0.071	0.42	1.74
	20.83	0.048	0.62	2.57
MoS_2_/WSe_2_	9.88	0.101	0.3	1.23
	13.98	0.071	0.42	1.74
	23.56	0.042	0.71	2.94
	27.66	0.036	0.83	3.44
MoS_2_/WSe_2_/WSe_2_	9.88	0.101	0.30	1.24
	13.99	0.072	0.42	1.74
	20.83	0.048	0.63	2.61
	27.84	0.036	0.84	3.48

## Data Availability

The original contributions presented in this study are included in the article. Further inquiries can be directed to the corresponding authors.

## Supplementary Materials

The following supporting information can be downloaded at: https://www.mdpi.com/article/10.3390/ma18071665/s1, Table S1. The SNR calculations of MoS_2_ and WSe_2_ Raman peaks; Table S2. The SNR calculations of MoS_2_/WSe_2_ and MoS_2_/WSe_2_/MoS_2_ heterostructure Raman peaks; Table S3. The corresponding frequency values of monolayer and few-layer MoS_2_ materials with different thicknesses at the Γ point; Table S4. The corresponding frequency values of monolayer and few-layer WSe_2_ materials at the Γ point; Table S5. The physical parameters of different materials for simulation modeling; Figure S1. The geometric morphology, size, and thickness parameters of the materials. (a) Bottom layer MoS_2_. (b) Middle layer WSe_2_. (c) Top layer MoS_2_. (d) MoS_2_/WSe_2_. (e) MoS_2_/WSe_2_/MoS_2_. (f) The side view of the MoS_2_/WSe_2_/MoS_2_ model on a large–area silicon substrate. (g) Top view of the model; Figure S2. The temperature change Δ*T* of the top MoS_2_ after irradiation by terahertz waves of 0.5–15 THz with THz power density variation; Figure S3. The temperature increase Δ*T* of the middle WSe_2_ after irradiation by terahertz waves of 0.5–15 THz with THz power density variation; Figure S4. The temperature change Δ*T* of the bottom MoS_2_ after irradiation by terahertz waves of 0.5–15 THz with THz power density variation; Figure S5. The simulation of temperature changes Δ*T* in each layer of the MoS_2_/WSe_2_/MoS_2_ heterojunction after irradiation by a single terahertz pulse (pulse duration Δ*t* = 120 s). (a) Bottom MoS_2_, (b) Middle WSe_2_, (c) Top MoS_2_, (d) MoS_2_/WSe_2_/MoS_2_ heterostructure.
