# Supplementary material for: Interactions of Terahertz Photons with Phonons of Two-Dimensional van der Waals MoS2/WSe2/MoS2 Heterostructures and Thermal Responses
Source: Materials (Basel). 2025 Apr 4;18(7):1665. doi: 10.3390/ma18071665 (PMC11990753; doi:10.3390/ma18071665)
Supplement: Supplementary file 1 [file materials-18-01665-s001.zip › materials-3549398-supplementary.pdf]

Supplementary Materials

Interactions of Terahertz Photons with Phonons of Two-dimensional van der Waals $MoS_2/WSe_2/MoS_2$ Heterostructures and Thermal Responses

Jingwen Huang[1], Ningsheng Xu[1,2], Yumao Wu[3], Xue Ran[3], Yue Fang[4], Hongjia Zhu[1], WeiliangWang[4], Huanjun Chen[1*], Shaozhi Deng[1*]

[1] State Key Laboratory of Optoelectronic Materials and Technologies, Guangdong Province Key Laboratory of Display Material and Technology, School of Electronics and Information Technology, Sun Yat-sen University, Guangzhou 510275, China.

[2] State Key Laboratory of Integrated Chips and Systems, Frontier Institute of Chip and System, Fudan University, Shanghai 200433, China.

[3] State Key Laboratory of Integrated Chips and Systems, School of Information Science and Technology, Fudan University, Shanghai 200433, China.

[4] Guangdong Province Key Laboratory of Display Material and Technology, Center for Neutron Science and Technology, School of Physics, Sun Yat-sen University, Guangzhou 510275, China.

* Correspondence: chenhj8@mail.sysu.edu.cn, stsdsz@mail.sysu.edu.cn.

Section 1: The wet method corrosion prepared the large area $MoS_2/WSe_2/MoS_2$ heterojunction

Chemicals used: 4% PMMA anisole solution, 2 mol/L KOH solution, deionized water, acetone (or anisole).

Transfer method:

1. Place large-area MoS_2 grown on the SiO_2/Si substrate on a heating stage, and drop 4-5 drops of PMMA anisole solution using a dropper.
2. Turn on the heating stage to 100°C to solidify the PMMA into a film.
3. Immerse the solidified SiO_2/Si substrate with PMMA/MoS_2 in a 2 mol/L potassium hydroxide (KOH) solution.

4. After soaking for about 2 hours, the PMMA film will naturally separate from the substrate.

5. Use tweezers to pick up the separated PMMA film and rinse it 3-5 times in deionized water to remove any residual KOH.

6. Use a blank high-resistivity silicon substrate to lift the PMMA film from below, allowing the large-area MoS_2 to adhere to the new substrate.

7. Place the lifted substrate vertically to allow residual water to flow to one corner, and let it air dry naturally.

8. After air drying, heat the substrate with MoS_2/PMMA on an 80 °C hot plate to improve the bonding between MoS_2 and the new substrate, preventing the sample from falling off during the next steps.

9. Immerse the prepared MoS_2/PMMA/high-resistivity silicon substrate in acetone to etch away the PMMA. This process typically requires changing the acetone 3-5 times, with each soak lasting about 30 minutes.

10. After removing the PMMA, dry and package the sample for storage.

To transfer another layer of large-area WSe_2 and the top layer of large-area MoS_2, simply repeat the above wet transfer process two more times.

Section 2: the SNR for the relatively weak peaks in each Raman spectrum

Table S1. the SNR calculations of MoS_2 and WSe_2 Raman peaks

MoS_2				WSe_2			
**Raman shift (cm^{-1})**	**I_{peak} Intensity**	**$I_{baseline}$ Intensity**	**SNR**	**Raman shift (cm^{-1})**	**I_{peak} Intensity**	**$I_{baseline}$ Intensity**	**SNR**
34.83	549.59	500.824	3.93	29.03	618.74	562.56	3.71
41.66	465.733	423.156	3.43	46.76	330.266	280.762	3.27
48.47	453.3	406.86	3.74	61.76	295.98	247.68	3.19
66.17	340.3	293.094	3.80	64.45	297.927	242.52	3.66
82.45	269.13	211.949	4.61	75.32	338.866	261.077	5.14
90.59	252.81	199.287	4.31	97.01	531.906	428.105	6.87

Table S2. the SNR calculation of MoS_2/WSe_2 and $MoS_2/WSe_2/MoS_2$ heterostructures Raman peaks

MoS_2/WSe_2				$MoS_2/WSe_2/MoS_2$			
**Raman shift (cm^{-1})**	**I_{peak} Intensity**	**$I_{baseline}$ Intensity**	**SNR**	**Raman shift (cm^{-1})**	**I_{peak} Intensity**	**$I_{baseline}$ Intensity**	**SNR**
35.85	441.562	379.03	4.94	35.85	326.133	271.955	4.28
41.3	374.036	323.63	3.99	41.3	265.859	219.524	3.66
49.48	302.852	258.279	3.52	52.21	231.351	191.384	3.16
56.29	306.795	237.089	5.51	69.89	184.036	138.387	3.61
64.46	257.467	213.376	3.49	73.97	180.452	140.612	3.15
68.53	241.044	197.871	3.41	86.18	156.764	116.414	3.19
72.61	250.38	192.12	4.61	97.02	173.608	132.953	3.22
92.95	251.04	207.35	3.45				

Section 3: The list of specific results of phonon spectrum calculation

Table S3. The corresponding frequency values of monolayer and few–layer MoS_2 materials with different thicknesses at the Γ point.

**MoS_2**	**point 1**	**point 2**	**point3**	**point 4**	**point 5**	**point 6**	**point 7**	**point 8**	**point 9**
1L	0	0	0	0	0	0	8.68	8.68	8.68
5L	0	0	0	0.42	0.42	0.71	8.24	8.24	8.25
**MoS_2**	**point 10**	**point 11**	**point 12**	**point 13**	**point 14**	**point 15**	**point 16**	**point 17**	**point 18**
1L	8.68	11.75	11.751	11.75	11.78	12.34	12.34	14.3	14.3
5L	8.25	11.14	11.14	11.14	11.21	11.85	11.88	13.62	13.66

Table S4. The corresponding frequency values of monolaye and few–layer WSe_2 materials at the Γ point.

**WSe_2**	**point 1**	**point 2**	**point3**	**point 4**	**point 5**	**point 6**	**point 7**	**point 8**	**point 9**
1L	0	0	0	0	0.04	0.04	5.14	5.14	5.14
5L	0	0	0	0.38	0.38	0.61	5.11	5.17	5.18
**WSe_2**	**point 10**	**point 11**	**point 12**	**point 13**	**point 14**	**point 15**	**point 16**	**point 17**	**point 18**
1L	5.14	7.32	7.32	7.32	7.35	7.35	7.35	9.13	9.14
5L	5.18	7.32	7.32	7.32	7.44	7.44	7.47	9.11	9.14

Section 4: The Thermodynamic simulation and calculation model parameters and part of the calculation results

Table S5. The physical parameters of different materials for simulation modeling

Materials	geometrical shape	Area cm^2	Thickness nm	Thermal conductivity W/(m·K)	Density g/cm^3	Specific heat capacity J/(g・K)	Thermal convection coefficient (W/(m^2 ・K))
**Top MoS_2**	quadrangle	1.49×10^{-5}	4.08	100	5.06	0.397	0.55
**Middle WSe_2**	quadrangle	2.47×10^{-5}	1.86	40	9.2	4	0.33
**bottom MoS_2**	pentagon	4.597×10^{-5}	3.4	100	5.06	0.397	0.18
**MoS_2/WSe_2**	quadrangle	0.687×10^{-5}	5.26	65.793	-	2.451	1.19
**$MoS_2/WSe_2/MoS_2$**	triangle	0.284×10^{-5}	9.34	77.396	-	1.754	2.86
**SiO_2**	square	1cm×1cm	300	1.4	2.18	0.742	5-15
**Si substrate**	square	1cm×1cm	500 um	130	2.329	1.418	10-25

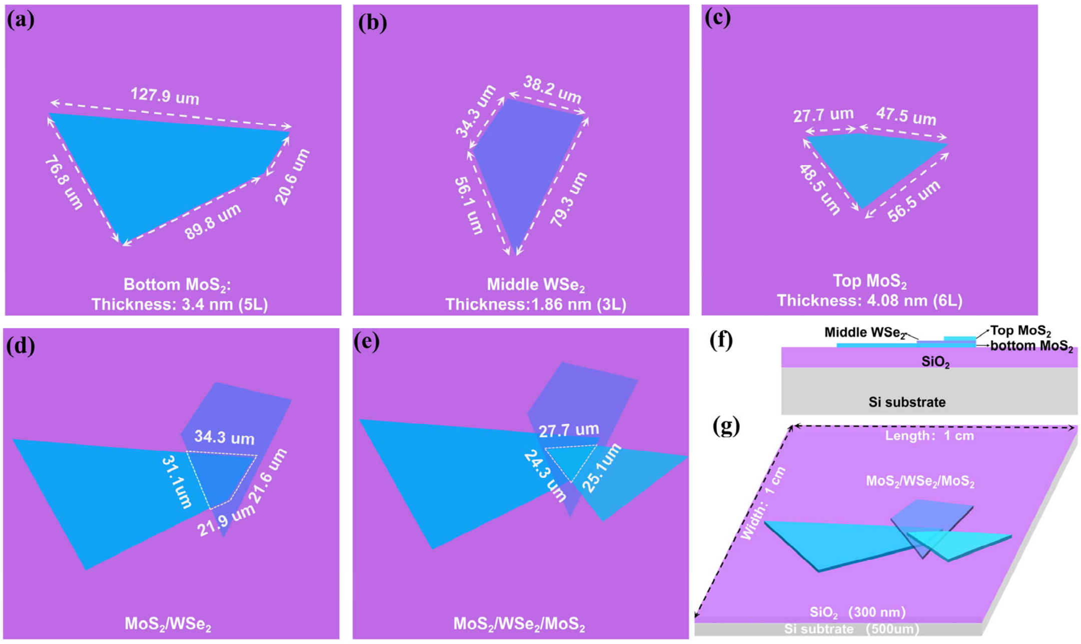

Figure S1. The geometric morphology, size, and thickness parameters of the materials. **(a)** Bottom layer MoS_2. **(b)** Middle layer WSe_2. **(c)** Top layer MoS_2. **(d)** MoS_2/WSe_2. **(e)** $MoS_2/WSe_2/MoS_2$. **(f)** The side view of the $MoS_2/WSe_2/MoS_2$ model on a large–area silicon substrate. (g) Top view of the model.

Figures s1a-c show the shape, size, and thickness of a single two-dimensional material. **Figure s1d** shows the shape and size of the transferred MoS_2/WSe_2 heterojunction and its overlapping region. **Figure s1e** shows the shape and size of the transferred $MoS_2/WSe_2/MoS_2$ heterojunction and its overlapping region. **Figure s1f and Figure s1g** show the side view and top view of the $MoS_2/WSe_2/MoS_2$ sample on a large area high resistance SiO_2/Si substrate (resistivity 20000 Ω • cm).

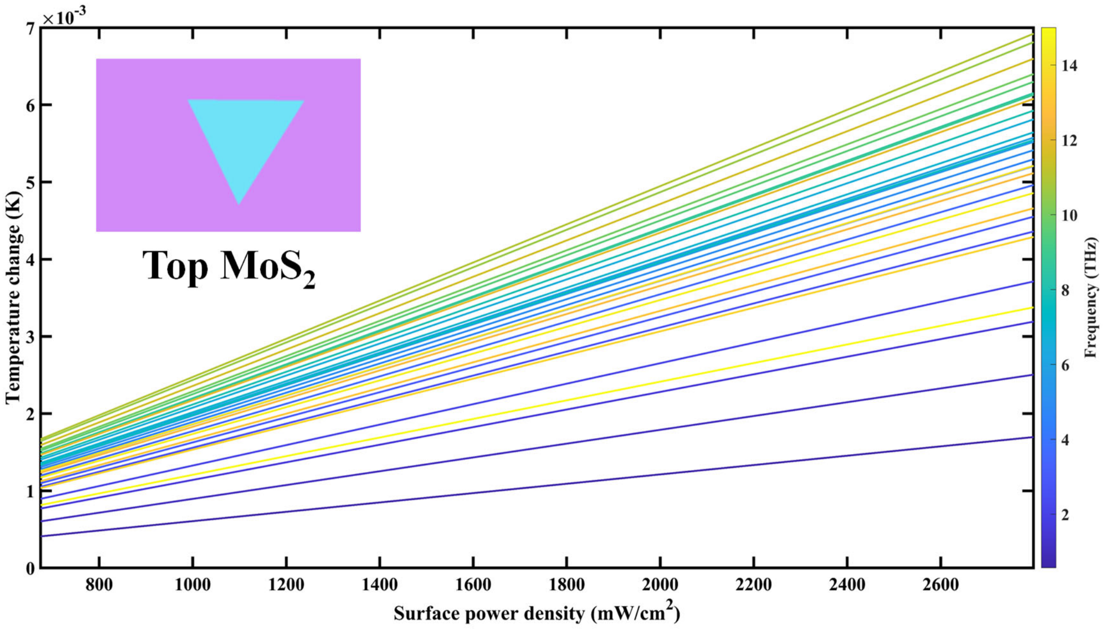

Figure S2 The temperature change ΔT of the top MoS_2 after irradiation by terahertz waves of 0.5–15 THz with THz power density variation.

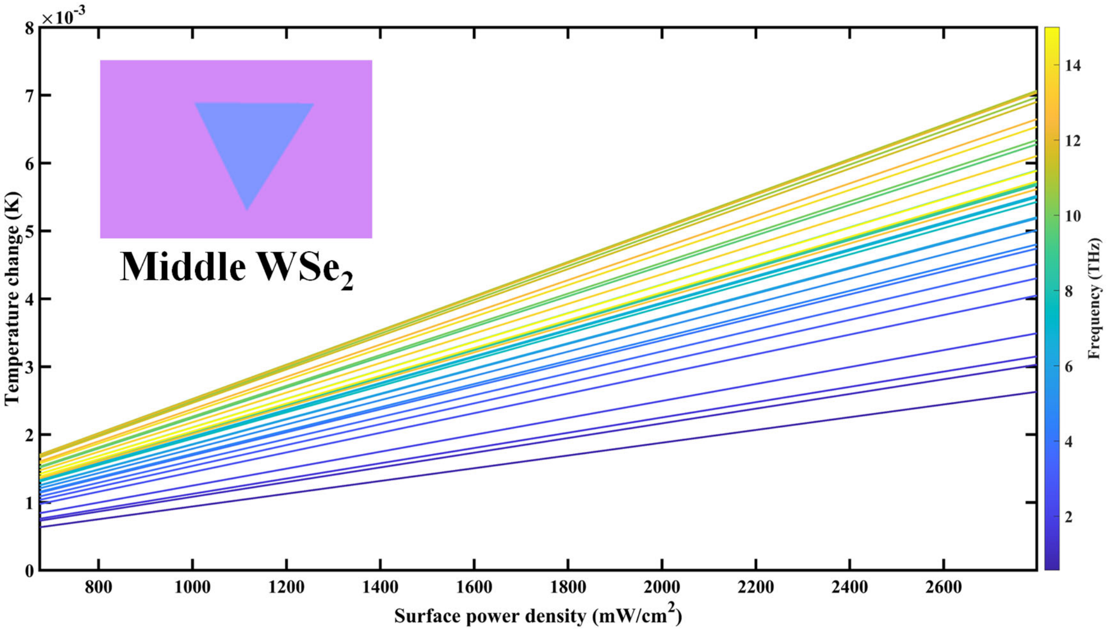

Figure S3. The temperature increases ΔT of the middleWSe$_2$ after irradiation by terahertz waves of 0.5–15 THz with THz power density variation.

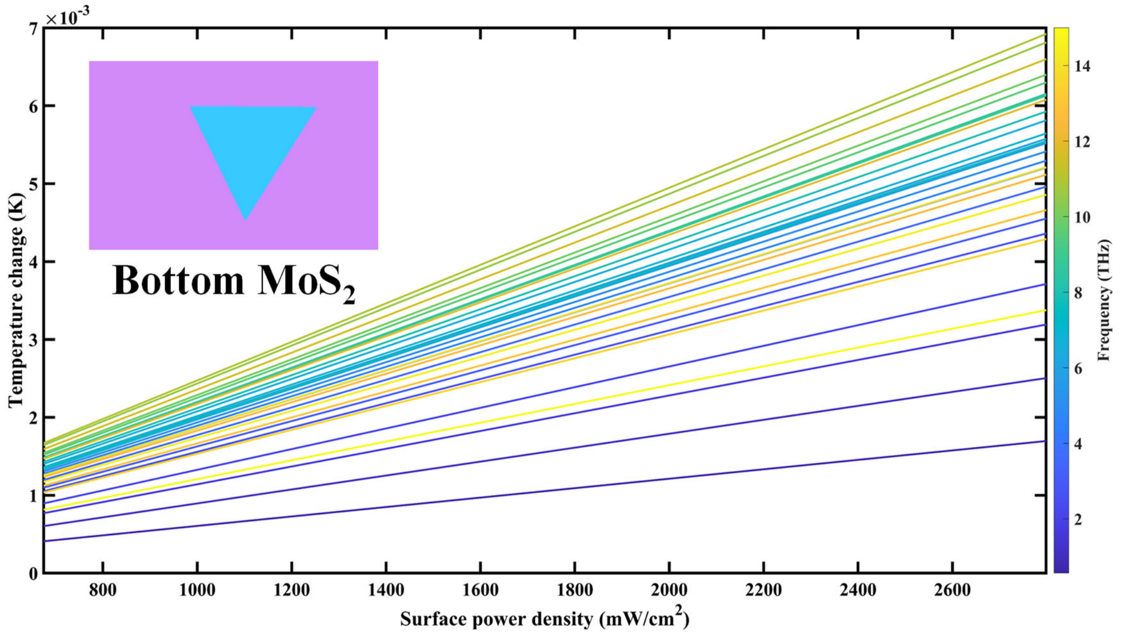

Figure S4. The temperature change ΔT of the bottom MoS_2 after irradiation by terahertz waves of 0.5–15 THz with THz power density variation.

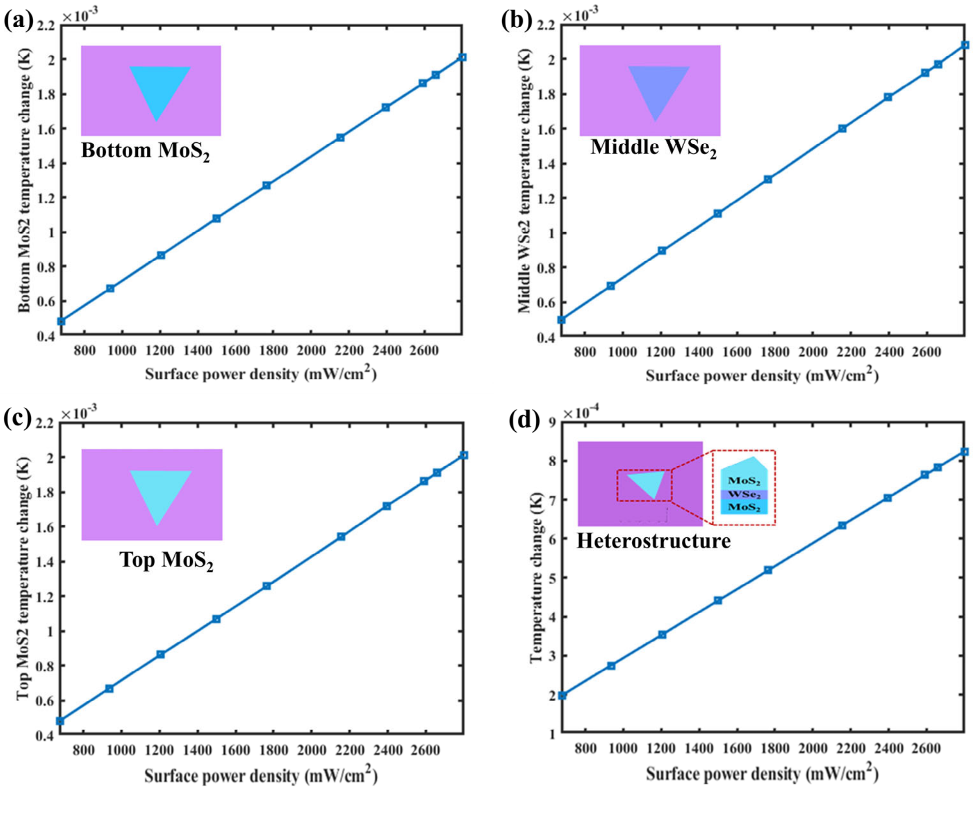

Figure S5. The simulation of temperature changes ΔT in each layer of the $MoS_2/WSe_2/MoS_2$ heterojunction after irradiation by a single terahertz pulse (pulse duration Δt = 120 s). **(a)** Bottom MoS_2, **(b)** Middle WSe_2, **(c)** Top MoS_2, **(d)** $MoS_2/WSe_2/MoS_2$ heterostructure.
